# Suitability and realism of the novel Fix for Life cadaver model for videolaryngoscopy and fibreoptic tracheoscopy in airway management training

**DOI:** 10.1186/s12871-020-01121-8

**Published:** 2020-08-15

**Authors:** Michael W. van Emden, Jeroen J. G. Geurts, Patrick Schober, Lothar A. Schwarte

**Affiliations:** 1 Department of Anatomy and Neurosciences, Amsterdam UMC, Vrije Universiteit, PO Box 7057, 1007 MB, De Boelelaan 1117, 1081 HV Amsterdam, The Netherlands; 2 Department of Anaesthesiology, Amsterdam UMC, Vrije Universiteit, De Boelelaan 1117, 1081 HV Amsterdam, The Netherlands

**Keywords:** Videolaryngoscopy, Fibreoptic intubation, Airway management training, Cadaver model

## Abstract

**Background:**

Videolaryngoscopy is increasingly advocated as the standard intubation technique, while fibreoptic intubation is broadly regarded as the ‘gold standard’ for difficult airways. Traditionally, the training of these techniques is on patients, though manikins, simulators and cadavers are also used, with their respective limitations. In this study, we investigated whether the novel ‘Fix for Life’ (F4L) cadaver model is a suitable and realistic model for the teaching of these two intubation techniques to novices in airway management.

**Methods:**

Forty consultant anaesthetists and senior trainees were instructed to perform tracheal intubation with videolaryngoscopy and fibreoptic tracheoscopy in four F4L cadaver models. The primary outcome measure was the verbal rating scores (scale 1–10, higher scores indicate a better rating) for suitability and for realism of the F4L cadavers as training model for these techniques. Secondary outcomes included success rates of the procedures and the time to successful completion of the procedures.

**Results:**

The mean verbal rating scores for suitability and realism for videolaryngoscopy was 8.3 (95% CI, 7.9–8.6) and 7.2 (95% CI, 6.7–7.6), respectively. For fibreoptic tracheoscopy, suitability was 8.2 (95% CI, 7.9–8.5) and realism 7.5 (95% CI, 7.1–7.8). In videolaryngoscopy, 100% of the procedures were successful. The mean (SD) time until successful tracheal intubation was 34.8 (30.9) s. For fibreoptic tracheoscopy, the success rate was 96.3%, with a mean time of 89.4 (80.1) s.

**Conclusions:**

We conclude that the F4L cadaver model is a suitable and realistic model to train and teach tracheal intubation with videolaryngoscopy and fibreoptic tracheoscopy to novices in airway management training.

## Background

Videolaryngoscopy (VLS) is an established standard airway technique, while the use of flexible fibreoptic or video tracheoscopy (FOT) is broadly regarded as the ‘gold standard’ when confronted with a difficult airway [1–3]. Traditionally, novice airway practitioners learn these techniques on patients in the operating room, though synthetic manikins or simulators are also being used, with their respective limitations [4]. The advantage of training outside the operating room is an environment free of risks to patients, and the option of constructing clinical scenarios not regularly encountered in practice [5]. However, mimicking the characteristics of human anatomy in synthetic manikins and simulators is difficult [6]. Human cadavers of persons who donated their body to science after death are potentially of added value in the training of VLS and FOT [7–9]. Such cadaver models reflect the variance in anatomy also encountered in real patients. However, the method of conservation of these cadaver models is crucial, because the traditional embalmment with large amounts of formaldehyde causes the tissues to be rigid and makes airway management training rather unrealistic. Recently, a new cadaver model has been described, embalmed with ‘Fix for Life’ (F4L), which trainee and specialist airway practitioners have found to be realistic and suited for teaching basic airway management techniques, e.g., mask ventilation [10]. In the present study, we investigated the suitability and realism of the F4L cadaver model for the training of two advanced video airway techniques, i.e., VLS and FOT for tracheal intubation.

## Methods

The study was approved by the biobank and ethics committee of the Amsterdam UMC, Vrije Universiteit, Amsterdam, the Netherlands. All data were collected in the anatomy laboratory of the department of Anatomy and Neurosciences.

### Participants

Forty consultant anaesthetists and senior trainees (4th and 5th year of the 5-year training program) were recruited to participate in the study. Inclusion criteria were familiarity with VLS and FOT for tracheal intubation, i.e., the participants are familiar with and have received training in these techniques. Due to formaldehyde being used at the anatomical facility, exclusion criteria were pregnancy and lactation. Before participating, all consultants and trainees gave written informed consent. Age, sex, number of years of professional experience and an estimation of the number of tracheal intubations with VLS and FOT (including anaesthetised and awake procedures) of the participants were recorded.

### F4L cadaver models

The four F4L cadavers used in this study were from body donors who donated their body to science after death through written consent, in accordance with Dutch legislation. Embalmment was performed within 24–72 h after demise. The cadavers were embalmed with the F4L embalmment fluids, according to the embalmment protocol for F4L fixation [11]. Basic characteristics of the cadavers (age at demise, sex, length, weight, body mass index) and morphometric predictors of difficult intubation (dental status, neck circumference, thyromental and sternomental distance) were recorded. The Cormack-Lehane grade of each F4L cadaver model was assessed in agreement by 2 senior consultant anaesthetists via direct laryngoscopy (Macintosh blade size 3) before the start of the study.

### Study protocol

Each participant performed the VLS and FOT procedures individually, with no other participants present in the room at the same time. The participants were instructed to first intubate the tracheas of the F4L cadaver models with the VLS (GlideScope®, Verathon Medical, Burnaby, Canada) with a size 3 blade. After completion of the VLS procedures on all cadaver models, the participants performed tracheal intubation via FOT (Ambu® aScope™ 4, Ambu A/S, Ballerup, Denmark, regular size, outer diameter 5.5 mm) on all four F4L cadaver models. Tracheal tubes were available in different sizes from 6.0 mm to 8.0 mm (Covidien™, Mansfield, MA). The procedures using VLS and FOT were performed in the same order for all participants on all four F4L cadavers. The participants were allowed to optimize the position of the head of the cadaver according to their own preferences (e.g., sniffing position or ramping). Any fluids present in the oropharyngeal cavity of the cadavers were suctioned before starting the procedures. One of the researchers present served as a ‘non-obstructive’ assistant to the participant to provide instruments (e.g., tracheal tube), or to apply jaw thrust or backward, upward, or rightward pressure (BURP) of the larynx, or other optimizing manoeuvres, if requested. For the FOT procedure, the participants were instructed to perform a nasotracheal intubation. The tracheal tube was allowed to be pre-fixed (‘loaded’) on the tracheoscope or pre-inserted through the nose of the cadaver model prior to the start of the FOT procedure, according to the preference of the participant. Lubricant was applied to the FOT device and tracheal tube, as required. Also, the participants were allowed to take their preferred position relative to the cadaver model (e.g., standing behind the ‘patient’ or next to the ‘patient’).

The time of the procedure (in seconds, [s]) was recorded for each intubation attempt. For the VLS intubation procedure, recording of time started when the tip of the VLS entered the mouth of the cadaver model and stopped when the tracheal tube was cuffed. Time of the FOT procedure was measured when the tip of the tracheoscope entered the cadaver’s nose (or the pre-inserted tracheal tube) and also stopped when the tracheal tube was cuffed. Active assistance upon request of the participant (e.g., jaw thrust) was recorded. Success of the VLS or FOT procedure was defined as a correct intubation of the trachea. In the VLS, correct placement of the tracheal tube was ascertained by direct view of the passing of the tube through the vocal cords on the GlideScope videoscreen by 2 of the present researchers. For the FOT procedures, correct placement of the tracheal tube was ascertained by confirming view of the carina on the aScope videoscreen. Failure of the procedures were additionally recorded if the participant resigned the task or if the time of the VLS procedure exceeded 5 min, or 10 min for the FOT procedure.

After completion of the intubation procedures on all cadaver models with VLS, and subsequently with FOT, the participants were asked to give an overall verbal rating score (VRS) for each technique [7, 10, 12]. The participants were asked to rate the F4L cadaver model for suitability as a training model to learn VLS or FOT (“Considering real-life patients as a reference, how suitable is the F4L cadaver model as a teaching model to teach novices the use of VLS or FOT with regard to the technical aspects?”). Thereafter, they were asked to score the model on realism (“Considering real-life patients as a reference, how realistic is the F4L cadaver model as a teaching model to teach novices the use of VLS or FOT with regard to look, feel and flexibility?”). The VRSs were given on a scale of 1 to 10 (1 = worst score, 10 = best score). Any relevant narrative feedback was also recorded.

### Outcome measures and statistical analysis

The primary outcome measures were the VRSs for suitability and for realism of the F4L cadaver as training model for VLS and FOT respectively. Secondary outcomes were success rates of the procedures, the time to successful intubation of the trachea and whether assistance was needed.

For this study we used a convenience sample of 40 consultant anaesthetists and senior trainees, and four F4L cadavers per participant. Statistical analysis was performed using SPSS, version 26 (IBM Corp, Armonk, NY). The mean VRSs are presented with calculated 95% confidence intervals (95% CI). Success rates of the VLS and FOT procedures are presented as proportions. Time, given in seconds, until successful intubation of the trachea is presented in mean with standard deviation (SD). The Mann-Whitney *U*-test was used to compare the VRSs given by consultant anaesthetists and the senior trainees. A *P* value < 0.05 was considered significant.

## Results

The participants included 26 consultant anaesthetists and 14 senior trainees with a mean (SD) professional experience of 11.7 (8.0) years. The male/female ratio was 20/20. Mean (SD) age was 40.2 (9.4) years. Self-estimated previous experience with VLS assisted tracheal intubations was < 20 in 5% of participants, and ≥ 20 in 95% of participants. Experience with FOT on patients was < 20 in 47.5% of participants, and ≥ 20 in 52.5% of participants. The characteristics of the 4 F4L cadaver models are presented in Table 1. All 40 participants completed all of the procedures on the 4 F4L cadaver models for a total of 160 VLS and 160 FOT assisted tracheal intubation attempts.

**Table 1:** Characteristics of the 4 Fix for Life (F4L) cadaver models

	Cadaver 1	Cadaver 2	Cadaver 3	Cadaver 4
Age at demise (y)	89	70	68	90
Sex	Male	Male	Female	Female
Weight (kg)	75	54	52	66
Length (m)	1.75	1.73	1.70	1.67
Body mass index (kg.m^− 2^)	24.5	18	18	23.7
Neck circumference (cm)	47	38	42	52
Thyromental distance (cm)	6.5	7.5	6	5.5
Sternomental distance (cm)	13.5	15	14	13.5
Dental status	Toothless	Toothless	Incomplete	Toothless
Cormack-Lehane grade	4	2	2	3

For suitability of training VLS, the mean VRS was 8.3 (95% CI, 7.9–8.6). For realism, the mean VRS was 7.2 (95% CI, 6.7–7.6). The suitability of the F4L cadaver model for FOT was rated with a mean VRS of 8.2 (95% CI, 7.9–8.5) and for realism, the mean VRS was 7.5 (95% CI, 7.1–7.8).

The results in proportion of successful procedures, time until successful completion and proportion of assistance needed are presented in Table 2.

**Table 2:** Results in Verbal Rating Scores (VRS) for suitability and for realism, success rates, time until successful completion, and requested assistance of the videolaryngoscopy (VLS) and flexible tracheoscopy (FOT) in the F4L cadaver model

	VLS	FOT
VRS suitability	8.3 (7.9–8.6)	8.2 (7.9–8.5)
VRS realism	7.2 (6.7–7.6)	7.5 (7.1–7.8)
Success rate	160 (100%)	154 (96.3%)
Time until completion; s	34.8 (30.9)	89.4 (80.1)
Assistance needed	22 (13.8%)	126 (78.8%)

Values are mean (95% confidence interval or standard deviation) or number (proportion).

No significant differences were observed in the mean (SD) VRSs given by consultant anaesthetists versus trainees respectively for suitability for VLS (8.5 [1.1] versus 7.9 [1.1], *P* = 0.190), realism for VLS (7.2 [1.4] versus 7.1 [1.5], *P* = 0.604), suitability for FOT (8.2 [1.0] versus 8.2 [0.8], *P* = 0.747), and realism for FOT (7.3 [1.1] versus 7.6 [1.0], *P* = 0.332). Additional comparative analyses of mean (SD) VRSs given by participants with < 20 and ≥ 20 FOT performed in patients respectively, revealed no significant differences in suitability for FOT (8.2 [1.1] versus 8.3 [0.8], *P* = 0.979) or for realism for FOT (7.7 [1.1] versus 7.2 [1.0], *P* = 0.161). For the mean (SD) VRSs given by participants with < 20 and ≥ 20 VLS performed in patients respectively, also no significant differences were observed in suitability for VLS (8.5 [0.7] versus 8.3 [1.2], *P* = 0.785), and realism for VLS (6.5 [0.7] versus 7.2 [1.4], *P* = 0.369).

The additional, narrative feedback provided by the participants was that the F4L cadaver model was ‘more rigid’, had a ‘paler or different colour’, and was ‘dryer’ in regard to real patients. Other remarks were the ‘setting differences’ (e.g., no beeping sounds of monitors), and the ‘not awake patient’.

**Fig. 1:**
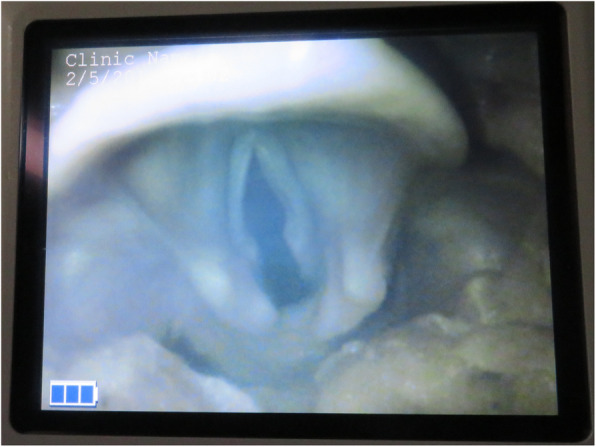
Laryngeal view with the videolaryngoscope

A typical example of the laryngeal view with VLS is presented in Fig. 1.

## Discussion

This is the first study to investigate the suitability and the realism of the novel F4L cadaver model as airway management training model for both VLS and FOT. Our results suggest that experienced airway practitioners regard the F4L cadaver as a suitable and realistic training model for both VLS and FOT procedures.

Different models for VLS and FOT training have been described, ranging from manikins, simulators [13–15], animals [16] and cadaver models [7, 9, 17]. Learning these airway techniques on different types of models outside the operating room could be effective, and time efficient [4, 5, 18]. In our study, the participants rated the F4L cadaver model high with regard to suitability and realism, considering real patients as a reference. This finding is comparable to an earlier study in which the F4L cadaver model was found to be a realistic and suitable model for more basic airway manoeuvres [10]. For example, suitability and realism as a teaching model for mask ventilation were scored as 7.2 and 7.0 respectively, which is consistent with our current findings. These scores are promising, and support the use of the F4L cadaver model for airway management training programmes. The results of the present study suggest extending the application spectrum of F4L cadavers from these more basic airway manoeuvres to the advanced airway manoeuvres, i.e., VLS and FOT. The F4L cadaver could be a useful asset to reduce the learning period of VLS and FOT procedures outside the operating theatre.

Simulation training has found a place in anaesthesia training programmes, although there is discussion about the degree of reality a simulation model should have [6, 19, 20]. Using the F4L cadaver in addition to simulators and manikins in airway management training could provide for optimal preparation of novice airway practitioners before executing these techniques on actual patients. In addition, experienced airway practitioners can refresh or optimize their technical skills outside the operating room. In the ever faster evolving market of novel airway devices, the F4L cadaver model may provide a safe ‘test field’ to test and train new devices before their first application in a real patient.

The success rate of intubation with VLS was 100%, which is within the range of reported success rates of 73–100% in a recent meta-analysis of Glidescope VLS [21]. Also, the mean time to successfully complete the VLS procedure did not exceed those previously reported [21, 22]. For the FOT procedures, the success rate was 96.3%, which is comparable with reported success rates from an analysis of 1612 fibreoptic intubation cases [23]. In this study, 93.9% of FOT procedures were successfully completed within 3 min. In our study on the F4L cadaver models, this was 90.9% within the same timeframe. In a recent manikin study, the success rate for nasotracheal intubation of the trachea with the Ambu aScope 3 was 95% [24]. In this report, the mean (SD) time for proper tracheal tube placement was 70 (33) seconds, which is on average 20 s faster compared to our measured mean time of approximately 90 s. However, only a single manikin was used in that study. In our study, occasionally oropharyngeal fluid collections were encountered during the procedure and were suctioned with the Ambu aScope, which adds time to the duration of the procedure. However, the F4L cadaver model probably resembles the clinical setting more closely, where blood or secretions may be encountered in the airway of patients.

There are some limitations to our study. The F4L cadaver model was not compared with other cadaveric preparations or manikins with regard to the performance of VLS and FOT, thus no conclusions can be drawn on its performance in comparison to these other models. We used only one type of VLS (Glidescope) and FOT (Ambu aScope 3) device, while there are multiple types available in practice. Our results are therefore not necessarily generalizable to other device types, but we did use broadly distributed devices, also mostly used in our hospital. We are aware that not every hospital has the availability of a cadaver lab, but a university hospital as ours serves also as a regional training centre, and airway courses are given to an (inter-)national public where the cadaver lab can be integrated in the curriculum. However, currently used standard formaldehyde-based fixation techniques result in very rigid cadavers, which are not useful for airway management training [10, 25]. While fresh frozen cadavers have the advantage that they are realistic after thawing, and are used in airway management training [26], continuing decomposition remains a major limitation. Ideally, a preservation technique would avert decomposition while at the same time preserve the natural characteristics of human tissue. The F4L preservation method appears to come quite close to this ideal as it provides for a flexible human cadaver model with comparable tissue quality as fresh frozen cadavers, yet without the disadvantage of ongoing decomposition. In contrast to the use of formaldehyde preserved cadaveric preparations, the necessary amount of formaldehyde in F4L cadaver models is much smaller, which reduces toxicity. A main advantage of formaldehyde preserved cadaveric preparation is the long duration these specimens can be used, usually for multiple years. In our experience, a well preserved F4L cadaver model can generally be used for a minimum of 2 years before the tissue quality diminishes. Due to these properties, the F4L cadaver is utilised at our facilities for the training and teaching of surgical procedures, and also for ultrasonography airway management courses in identifying anatomical structures in patients (e.g. the cricothyroid membrane for front-of-neck access) [27]. Regarding our field of interest, the F4L cadaver model can also be used to learn to handle different airway devices, while providing a rather realistic anatomical view. For this first study of VLS and FOT in F4L cadaver models, we selected cadavers with rather normal habitus and morphology. For follow-up studies and our airway training courses, cadavers with more challenging characteristics (e.g., obesity) can also be selected and preserved.

## Conclusions

In conclusion, our results suggest that the F4L cadaver model is a realistic and suitable model for the training and teaching of VLS and FOT airway manoeuvres to novices in airway management. We see potential for the F4L cadaver model to be incorporated in airway training curricula.

## Data Availability

The datasets used and/or analysed during the current study are available from the corresponding author on reasonable request.

## Abbreviations

CI: Confidence Interval
F4L: Fix for Life
FOT: Fibreoptic tracheoscopy
SD: Standard Deviation
VLS: Videolaryngoscopy
VRS: Verbal Rating Scale
